# AZGP1P2/UBA1/RBM15 Cascade Mediates the Fate Determinations of Prostate Cancer Stem Cells and Promotes Therapeutic Effect of Docetaxel in Castration-Resistant Prostate Cancer via TPM1 m6A Modification

**DOI:** 10.34133/research.0252

**Published:** 2023-10-17

**Authors:** Hong Wang, Ji Liu, Xiaojun Zhu, Bin Yang, Zuping He, Xudong Yao

**Affiliations:** ^1^ Department of Urology, Shanghai Tenth People’s Hospital, School of Medicine, Tongji University, Shanghai, China.; ^2^ Urologic Cancer Institute, School of Medicine, Tongji University, Shanghai, China.; ^3^ Department of Urology Surgery, The Affiliated Hospital of Inner Mongolia Medical University, Hohhot, China.; ^4^ The Key Laboratory of Model Animals and Stem Cell Biology in Hunan Province, School of Medicine, Hunan Normal University, The Engineering Research Center of Reproduction and Translational Medicine of Hunan Province, Changsha, China.; ^5^ Shanghai Key Laboratory of Reproductive Medicine, Shanghai Jiao Tong University School of Medicine, Shanghai, China.

## Abstract

Prostate cancer (PCa) is a common malignant tumor with high morbidity and mortality worldwide. The prostate cancer stem cell (PCSC) model provides novel insights into the pathogenesis of PCa and its therapeutic response. However, the roles and molecular mechanisms of specific genes in mediating fate decisions of PCSCs and carcinogenesis of PCa remain to be elusive. In this study, we have explored the expression, function, and mechanism of AZGP1P2, a pseudogene of AZGP1, in regulating the stemness and apoptosis of PCSCs and treatment resistance of docetaxel in castration-resistant prostate cancer (CRPC). We revealed that AZGP1P2 was downregulated in CRPC cell lines and PCSCs, while it was positively associated with progression-free interval. Upregulation of the AZGP1P2 enhanced the sensitivity of docetaxel treatment in CRPCs via inhibiting their stemness. RNA pull-down associated with mass spectrometry analysis, co-immunoprecipitation assay, and RNA immunoprecipitation assay demonstrated that AZGP1P2 could bind to UBA1 and RBM15 as a “writer” of methyltransferase to form a compound. UBA1, an E1 ubiquitin-activating enzyme, contributed to RBM15 protein degradation via ubiquitination modification. Methylated RNA immunoprecipitation assay displayed that RBM15 controlled the mRNA decay of TPM1 in m6A methylation. Furthermore, a xenograft mouse model and patient-derived organoids showed that the therapeutic effect of docetaxel in CRPC was increased by AZGP1P2 in vivo. Collectively, these results imply that AZGP1P2 mediates the stemness and apoptosis of PCSCs and promotes docetaxel therapeutic effect by suppressing tumor growth and metastasis via UBA1/RBM15-mediated TPM1 mRNA decay in CRPC.

## Introduction

Prostate cancer (PCa) has been reported as the leading malignant tumor threatening men’s health [1]. At the early stage, surgery or androgen deprivation therapy (ADT) may achieve satisfactory efficacy, whereas advanced PCa patients inevitably progress into castration-resistant prostate cancer (CRPC). At this stage, chemotherapy drugs, e.g., docetaxel, have been chosen as the first-line treatment [2]. However, after an initial period of response to therapy, most of the PCa patients inexplicably progress into the chemotherapy-resistant stage [3]. Cancer stem cells (CSCs), also known as tumor-initiating cells (TICs), possess a variety of capacities, including self-renewal and differentiation, which are associated with therapeutic resistance and recurrence [4,5]. It is of great significance to uncover the mechanisms of chemotherapy resistance to achieve the resumption of chemotherapy sensitivity and provide an ideal strategy for the treatment of CRPC.

N6-methyladenosine (m6A) frequently occurs in the N6-site of adenosine, and it has mainly been modified at eukaryotic mRNA or non-coding RNA. The m6A methylation mediates RNA epigenetic modification, and it participates in numerous RNA processes, including transcription, maturation, translation, stabilization, and degradation, reflecting that m6A methylation plays critical roles in many physiological activities [6,7]. It has been shown that dysregulation of m6A modification results in various diseases, e.g., tumorigenesis, heart failure, Alzheimer’s disease, and obesity [8]. Critical enzymes, including “writers”, “erasers”, and “readers”, participate in catalysis and reversibility of m6A modification in physiological processes [6,7]. Chemotherapy is effective for most malignant tumors, whereas most patients eventually become chemotherapy resistant. Remarkably, growing evidence indicates that m6A modification of numerous genes is related to cancer treatment resistance [9].

Ubiquitination and deubiquitination have been reported to be involved in a variety of cell biological processes [10]. The ubiquitin–proteasome system, including ubiquitin-activating enzymes (E1), ubiquitin-conjugating enzymes (E2), ubiquitin ligases (E3), and 26 S proteasome and deubiquitinating enzymes (DUB), maintains the cellular protein homeostasis [11]. Disorder of the modification process leads to many dysfunctional biological processes, e.g., cell cycle progression, DNA damage response, gene transcription, and protein translation. In brief, the ubiquitination process is triggered by formation of the E1-ubiquitin thioester component at the active Cys residue site of E1 under ATP-dependent reactions. After E1-ubiquitin is transferred to E2 in a thioester linkage conjugate manner, E3 brings the ubiquitin of the E2-ubiquitin conjugate to the Lys residues site within the relevant substrate proteins at the C-terminal tail of ubiquitin by recognizing their specific motifs. The target proteins are transferred to the 26 S proteasome complex for degradation or DUB enzymes for reversing the ubiquitination process by removing ubiquitin [12].

AZGP1P2, a pseudogene of AZGP1, is located at chromosome 7, and it encodes Zinc-α2-glycoprotein (ZAG). AZGP1 has been demonstrated in various physiological and pathological processes, including glucose metabolism, lipolysis, epilepsy, and cancer [13–15]. In CRPC, the low expression level of AZGP1 has been shown to be associated with increased mortality, and AZGP1 has been regarded as an independent predictor of biochemical relapse following radical prostatectomy [16–18]. Nevertheless, it remains unclear whether AZGP1P2 participates in the biological process of CRPC and its underlying mechanisms have not been elucidated. In the current study, we have demonstrated, for the first time, the function and mechanisms of AZGP1P2 in controlling the treatment resistance of docetaxel in CRPC by regulating the stemness of prostate cancer stem cells (PCSCs). We found that AZGP1P2 enhanced docetaxel therapeutic effect by suppressing the growth and metastasis of CRPC through TPM1 mRNA decay by UBA1/RBM15 in CRPCs. This study thus provides a novel mechanism underlying the carcinogenesis of CRPC, and it could offer a new approach for gene therapy of CRPC.

## Results

### AZGP1P2 is expressed at lower levels in CRPC cells and PCSC-like cells

**Fig. 1.**
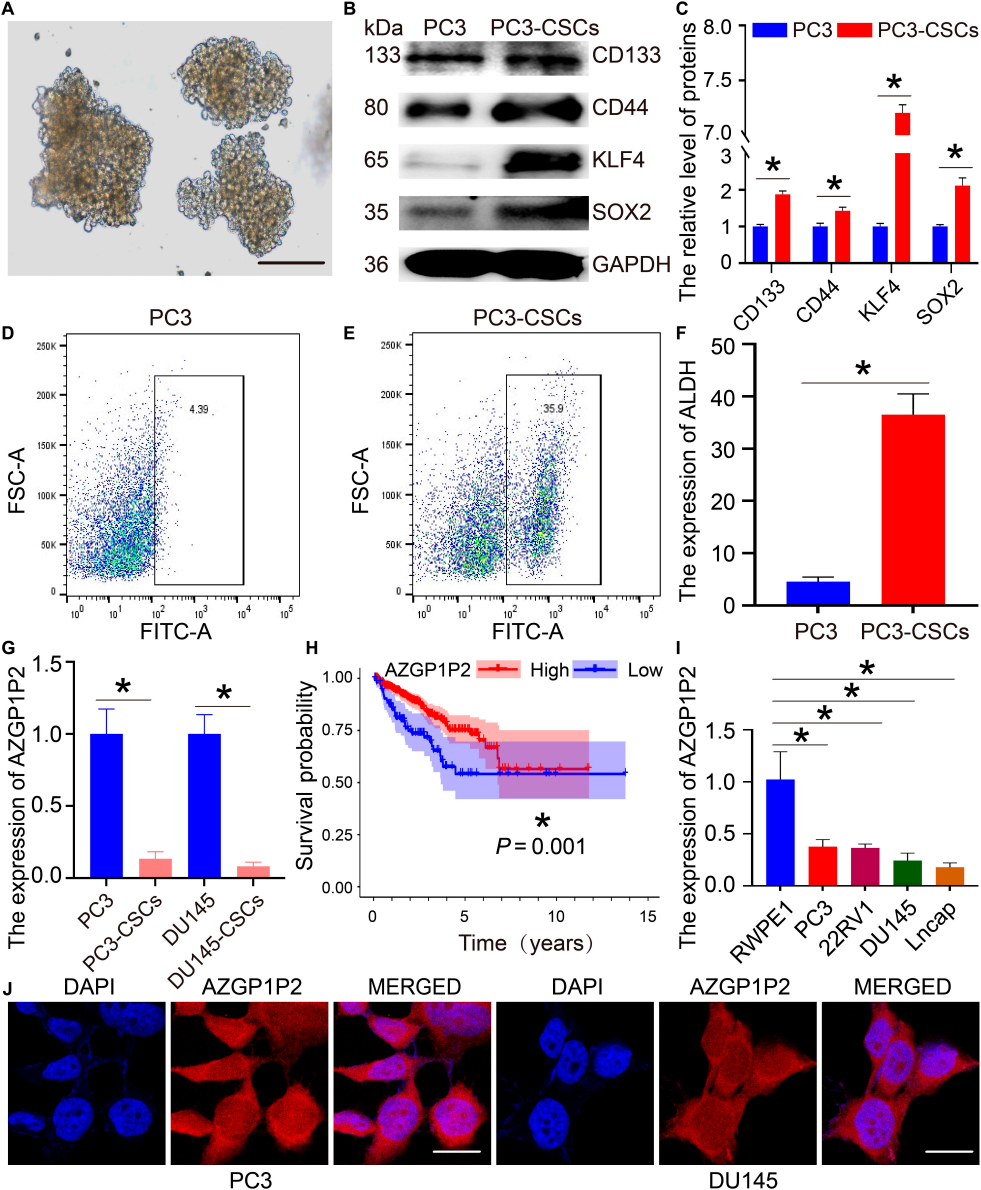
AZGP1P2 is expressed at lower levels in CRPC cells and PCSC-like cells. (A) The enrichment of prostate cancer stem (PCSC)-like cells derived from PC3 cells via serum-free suspension culture. The sphere with a diameter of more than 100 μm was regarded as stem-like cells. Scale bars in A = 100 μm. (B) Western blots revealed the protein expression of stem cell markers, including CD133, CD44, KLF4, and SOX2, in PC3 and PC3-CSCs. (C) The relative expression of CD133, CD44, KLF4, and SOX2 in PC3 cells and PC3-CSCs after normalization to the signals of their loading control GAPDH. (D to F) Flow cytometry analysis showed the ALDH expression level in PC3 (D) and PC3-CSCs (E). (G) Real-time PCR showed the expression of AZGP1P2 mRNA in PC3, PC3-CSCs, DU145, and DU145-CSCs. (H) The correlation of AZGP1P2 expression and progression-free interval of 92 CRPC patients from the TCGA database. (I) Real-time PCR evaluated the expression of AZGP1P2 in PC3, DU145, 22RV1, Lncap, and RWPE-1 cell lines. (J) FISH assay showed the subcellular localization of AZGP1P2 in PC3 and DU145 cells. Scale bars in J = 10 μm. All the results were presented from 3 independent experiments. * denoted statistically significant differences (*p* < 0.05).

CD133, CD44, KLF4, SOX2, ALDH1A1, and other proteins have been regarded as the hallmarks for PCSCs [19]. In the present study, we chose CD133, CD44, KLF4, SOX2, and ALDH to assess the stemness of PCSCs. After being cultured in the defined SFM (serum-free medium), the remaining PC3 cells formed the suspended spheres of cells (Fig. 1A). Western blots demonstrated that CD133, CD44, KLF4, and SOX2 were expressed at high levels in stem-like cells derived from the PC3 cells (Fig. 1B and C). Furthermore, flow cytometry revealed that PCSC-like cells (PC3-CSCs) isolated using SFM expressed ALDH at a higher level than the PC3 cells (36.53 ± 3.949 vs. 4.57 ± 0.8626) (Fig. 1D to F). Real-time polymerase chain reaction (PCR) showed that the transcript of AZGP1P2 was downregulated in PCSC-like cells (PC3-CSCs and DU145-CSCs) derived from PC3 and DU145 cells (Fig. 1G), suggesting that AZGP1P2 plays a vital role in the biological behavior of PCSC-like cells and CRPC. We found that high-AZGP1P2 patients had the better progression-free interval (PFI) (Fig. 1H). Real-time PCR displayed that AZGP1P2 was expressed at lower levels in PCa cell lines, including PC3, DU145, Lncap, and 22RV1, compared with human prostate epithelial cells RWPE-1 (Fig. 1I). Fluorescence in situ hybridization (FISH) assay further demonstrated that AZGP1P2 was located at the cytoplasm and nuclei of CRPC cells (Fig. 1J), reflecting that AZGP1P2 acts at the cytoplasm and nuclei of these cells. Together, these data imply that AZGP1P2 is expressed at lower levels in CRPC cells and PCSC-like cells.

### AZGP1P2 increases the sensitivity of docetaxel chemotherapy in CRPC

**Fig. 2.**
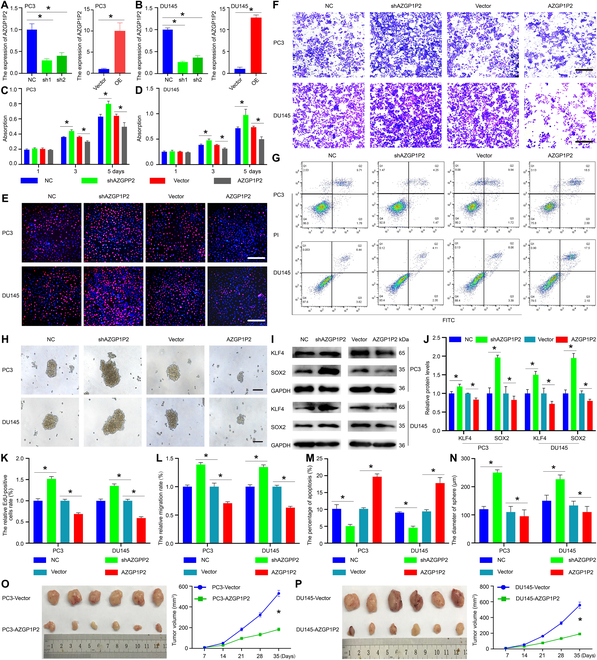
AZGP1P2 enhances the therapeutic effect of docetaxel in PCa in vitro and in vivo. (A and B) Knockdown and overexpression of AZGP1P2 in PC3 and DU145 cells by AZGP1P2 shRNA and lentivirus transfection. (C and D) CCK-8 assay showed the growth curve of PC3 and DU145 cells treated with docetaxel after knockdown and overexpression of AZGP1P2. (E) EdU assay illuminated the EdU-positive cells in PCa cells treated with docetaxel after knockdown and overexpression of AZGP1P2. Cell nuclei were counterstained with DAPI. The percentages of EdU-positive cells were counted out of at least of 500 cells from 3 independent experiments. Scale bars in E = 100 μm. (F) The migration abilities of CRPC cells were analyzed by Transwell assays of PC3 and DU145 cells treated with docetaxel after AZGP1P2 knockdown or overexpression. Scale bars in F = 100 μm. (G) Annexin V-FITC/PI assay showed the percentages of apoptosis in PC3 and DU145 cells treated with docetaxel after knockdown or overexpression of AZGP1P2. (H) Sphere formation assay revealed that knockdown or overexpression of AZGP12P changed the stemness of CRPC. Scale bars in H = 100 μm. (I) Western blots displayed the protein expression of KLF4 and SOX2 after knockdown or overexpression of AZGP1P2 in PC3 and DU145 cells. (J) The relative expression of KLF4 and SOX2 in CRPC cells after AZGP1P2 knockdown or overexpression normalized to the signals of their respective loading control GAPDH. (K to N) The changes of relative EdU-positive cells, migration rate, percentages of apoptosis, and diameters of sphere in CRPC cells after knockdown or overexpression of RBM15 or AZGP1P2. (O and P) Overexpression of AZGP1P2 in CRPC cells remarkably increased the sensitivity of docetaxel in the xenograft mice. Overexpression of AZGP1P2 significantly reduced volumes of tumors in the xenograft mice treated with docetaxel. All the results were presented from 3 independent experiments. * indicated statistically significant differences (*p* < 0.05).

We utilized shRNAs and recombinant pLenO-GTP-AZGP1P2-overexpression plasmids to examine the effect of AZGP1P2 on the function of docetaxel chemotherapy sensitivity of CRPC. Real-time PCR demonstrated that AZGP1P2 shRNA1-2 and AZGP1P2-overexpression plasmid remarkably decreased and enhanced the mRNAs of AZGP1P2 in CRPC cells, respectively (Fig. 2A and B and Fig. S1), while there was no obvious change in the parent gene AZGP1 and parallel AZGP1P1 in CRPC cells. We chose the shRNAs and vectors to explore the function of AZGP1P2 in the treatment effect of docetaxel in CRPC. The CCK-8 proliferation assay revealed that overexpression of AZGP1P2 significantly upregulated the sensitivity of docetaxel chemotherapy in 2 types of CRPC cells (PC3 and DU145), whereas AZGP1P2 silencing remarkably downregulated the sensitivity of these 2 cell lines (Fig. 2C and D). The 5-Ethynyl-2′-deoxyuridine (EdU) assay showed that the percentages of EdU-positive cells were increased by AZGP1P2 knockdown in CRPC cells treated with docetaxel and decreased by AZGP1P2 overexpression in PC3 and DU145 cells (Fig. 2E to K). Transwell assay demonstrated that the migration ability was upregulated in CRPC cells treated with docetaxel after AZGP1P2 knockdown, and it was downregulated by AZGP1P2 overexpression (Fig. 2F and L). Furthermore, Annexin V-fluorescein isothiocyanate (FITC)/propidium iodide (PI) assay showed that AZGP1P2 silencing resulted in the decrease in the percentages of apoptosis in PC3 cells (from 10.17% ± 1.26% to 5.07% ± 0.51%) and DU145 cells (from 9.07% ± 0.25% to 4.60% ± 0.46%) treated with docetaxel (Fig. 2G to M). In contrast, AZGP1P2 overexpression led to an increase in the sensitivity of docetaxel chemotherapy and the percentages of apoptotic cells (from 10.17% ± 0.35% to 19.67% ± 0.86%) in PC3 cells and DU145 cells (from 9.43% ± 0.50% to 17.80% ± 1.65%) (Fig. 2G to M). After being cultured in serum-free medium for 2 weeks, the survival CRPC cells formed sphere colony containing capable stem-like cells. As shown in Fig. 2H, the diameter of the sphere derived from AZGP1P2 silencing in CRPC cells was significantly increased (119.81 ± 8.12 μm vs. 248.00 ± 12.53 μm in PC3 cells; 148.33 ± 18.61 μm vs. 225.00 ± 15.40 μm in DU145 cells) (Fig. 2H and N). By contrast, the diameter was reduced by AZGP1P2 overexpression in CRPC cells (107.67 ± 19.50 μm vs. 95.67 ± 21.39 μm in PC3 cells; 131.33 ± 15.40 vs. 107.33 ± 20.01 μm in DU145 cells) (Fig. 2H and N). Western blots further demonstrated that the levels of KLF4 and SOX2, markers for the PCSCs [20], were significantly enhanced or decreased by AZGP1P2 silencing and overexpression, respectively (Fig. 2I and J). Considered together, these results indicate that AZGP1P2 overexpression reduces the stemness of CRPC cells and promotes the chemotherapy sensitivity of docetaxel in these cells. Furthermore, in vivo assay with the subcutaneous xenograft mouse model showed that AZGP1P2 overexpression decreased the tumor growth of CRPC cells treated with docetaxel (Fig. 2O and P and Fig. S2), which illustrates that AZGP1P2 plays an important role in improving the sensitivity of docetaxel chemotherapy in CRPC cells.

### AZGP1P2/UBA1/RBM15 complex induces ubiquitination and proteasomal degradation of RBM15 in CRPC cells

**Fig. 3.**
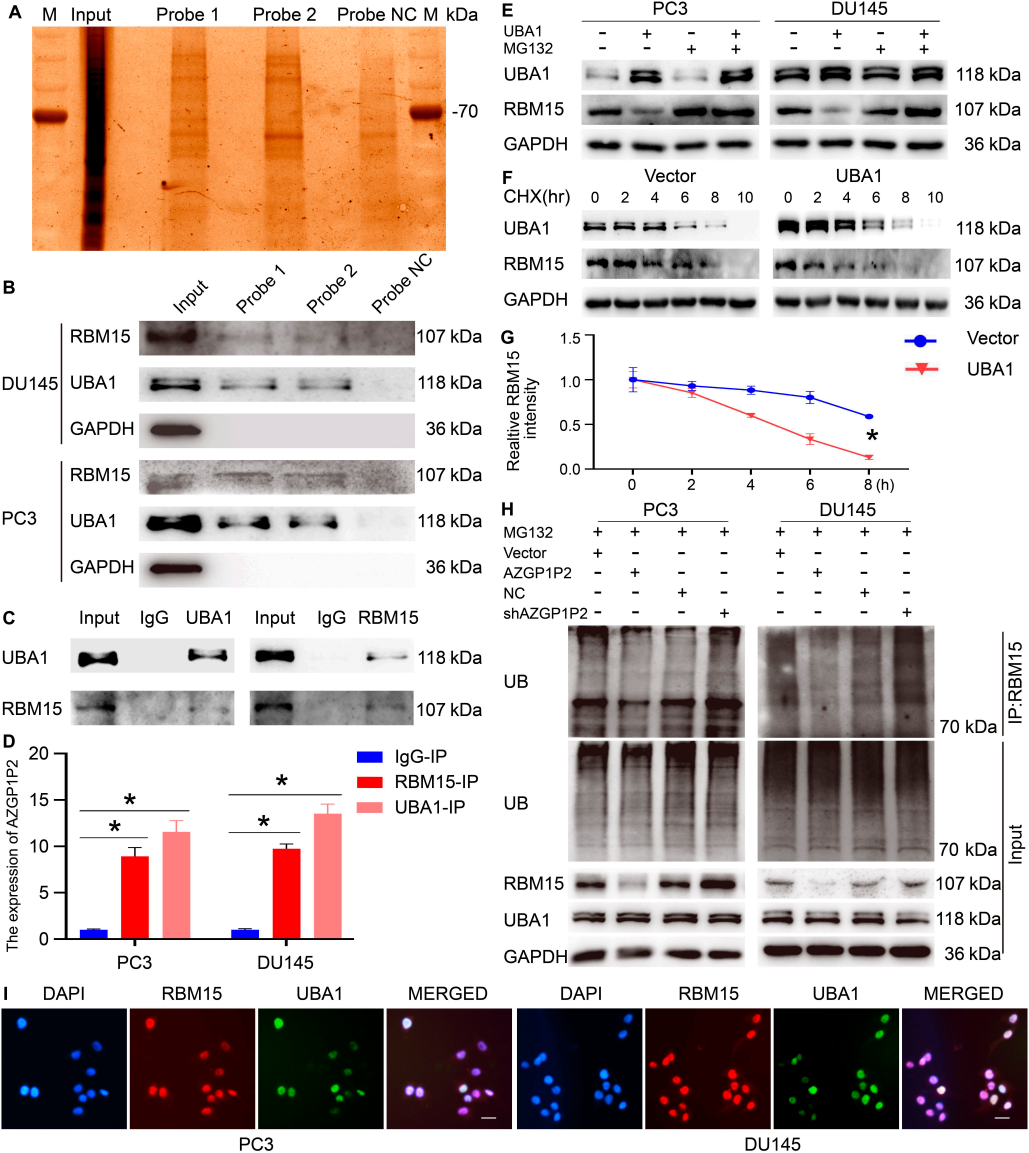
AZGP1P2 interacts with RBM15 and UBA1 to form a complex, which induces ubiquitination and proteasomal degradation of RBM15 in CRPC cells. (A and B) AZGP1P2 interacted with RBM15 and UBA1 by the RNA pull-down assay, mass spectrometry (MS), and Co-IP and Western blots in PCa cells. (C) Co-IP assay showed that the interaction of RBM15 and UBA1 utilizing RBM15 and UBA1 plasmid transfection in PC3 cells. (D) RIP assays showed the binding of RBM15 and UBA1 to AZGP1P2. (E) Ectopic expression of UBA1 reduced the RBM15 protein levels in PC3 and DU145 cells, which was enhanced by the proteasome inhibitor MG132 (20 μM). The ubiquitination level of RBM15 was mediated by UBA1 in PC3 and DU145 treated with MG132 (20 μM) for 6 h before being harvested for the Co-IP assays using the anti-RBM15 antibody. (F and G) The half-lives of RBM15 were shortened by RBM15 overexpression. The cells were harvested at different time points treated with 20 μM of cycloheximide (CHX). (H) The relative expression of RBM15 and UBA1 in PC3 and DU145 cells after normalization to the signals of their respect loading control. (I) Double immunofluorescence showed the subcellular localization of RBM15 and UBA1 in PCa cells. Scale bars in H = 10 μm. All the results were presented from 3 independent experiments. * denoted statistically significant differences (*p* < 0.05).

It has been reported that AZGP1P2 works via an association with RNA binding proteins [21]. To decipher the underlying molecular mechanism of AZGP1P2 in regulating docetaxel chemotherapy sensitivity of CRPC cells, RNA pull-down assay was performed (Fig. 3A). Mass spectrometry (MS) analyses and Western blots revealed that AZGP1P2 could bind to RBM15 and UBA1 of CRPC cells to form a complex (Fig. 3B). Furthermore, co-immunoprecipitation (Co-IP) and Western blots demonstrated an interaction of RBM15 and UBA1 in PC3 cells (Fig. 3C, Table S3 for AZGP1P2 probe binding proteins, and Table S4 for NC probe binding proteins). Besides, RNA immunoprecipitation (RIP) assay demonstrated that endogenous AZGP1P2 was co-immunoprecipitated with RBM15 and UBA1 (Fig. 3D). Together, these results imply that AZGP1P2, RBM15, and UBA1 could form a complex to regulate the chemotherapy sensitivity of docetaxel in CRPC. Since UBA1 is a component of the ubiquitin-activating enzyme, we asked whether UBA1 regulated the protein stability of RBM15 through their physical interaction. After transfecting UBA1-overexpression plasmid into PC3 and DU145 cells, we found that the expression level of the RBM15 was significantly reduced (Fig. 3E), and notably, this reduction was rescued by the proteasome inhibitor MG132 (Fig. 3E). Cycloheximide-chase assay revealed that AZGP1P2 overexpression diminished the half-life of RBM15 in PC3 and DU145 cells (Fig. 3F and G). Additionally, Co-IP assay indicated that endogenic AZGP1P2 regulated the ubiquitination level of RBM15 (Fig. 3H), reflecting an essential role of the AZGP1P2 in ubiquitination modification of RBM15. AZGP1P2 was negatively associated with the expression of RBM15 (Fig. 3H and Fig. S3), while AZGP1P2 overexpression or knockdown did not alter the UBA1 expression level (Fig. 3H and Fig. S3). Collectively, these data reflect that AZGP1P2 interacts with UBA1 that reduces RBM15 expression level via the RBM15 protein ubiquitination-mediated degradation. Double immunofluorescence staining demonstrated that the interaction of RBM15 and UBA1 occurred in the cell nuclei of PC3 and DU145 cells (Fig. 3I). Taken together, these data suggest that AZGP1P2, RBM15, and UBA1 formed a complex to regulate the chemotherapy sensitivity of docetaxel in PC3 and DU145 cells, probably via the mechanism that UBA1 activates the ubiquitination-mediated degradation of RBM15.

### RBM15 diminishes the docetaxel chemotherapy sensitivity in CRPC

**Fig. 4.**
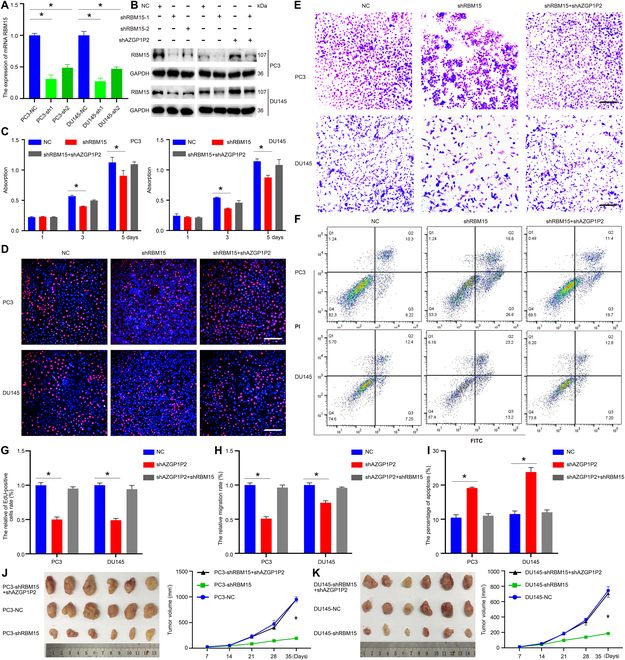
Knockdown of RBM15 decreases the docetaxel chemotherapy sensitivity in CRPC cells. (A) Real-time PCR showed RBM15 mRNA level by RBM15 shRNAs in PC3 and DU145 cells. (B) Western blots revealed the RBM15 protein level by RBM15 shRNAs in PC3 and DU145 cells. (C) CCK-8 assay illustrated the proliferation of PC3 and DU145 cells treated with docetaxel after RBM15 and AZGP1P2 silencing. (D) EdU assay showed the EdU-positive cells of PC3 and DU145 cells treated with docetaxel after RBM15 and AZGP1P2 silencing. Scale bars in D = 100 μm. (E) The migration abilities of CRPC cells were analyzed by Transwell assays treated with docetaxel after RBM15 and AZGP1P2 knockdown. Scale bars in E =100 μm. (F) Annexin V-FITC/PI assay showed the percentages of apoptotic cells treated with docetaxel after knockdown of RBM15 and AZGP1P2 in CRPC cells. (G to I) The changes of relative EdU-positive cells, migration rates, and apoptotic cells in CRPC cells after knockdown or overexpression of RBM15 or AZGP1P2. (J and K) Knockdown of RBM15 in CRPC cells obviously decreased prostate tumor growth in the xenograft mice treated with docetaxel. Results were presented from 3 independent experiments. * indicated statistically significant differences (*p* < 0.05).

We utilized shRNAs to investigate the effect of RBM15 on the function of docetaxel chemotherapy sensitivity of CRPC. Real-time PCR and Western blots revealed that transcription and translation of RBM15 were decreased by RBM15 shRNA1-2 in PC3 and DU145 cells (Fig. 4A and B). Furthermore, the downregulation of RBM15 could be rescued by shAZGP1P2 (Fig. 4B). CCK-8 (Fig. 4C), EdU (Fig. 4D and G), and Transwell assays (Fig. 4E and H) showed that the abilities of proliferation and migration were decreased by RBM15 shRNA in CRPC cells treated with docetaxel, and the decreases were rescued by AZGP1P2 knockdown (Fig. 4D, E, G, and H). Annexin V-FITC/PI assay showed that after RBM15 knockdown, the percentages of apoptotic cells were increased from 10.47% ± 0.85% to 19.1%7 ± 0.25% in PC3 cells, and from 11.50% ± 0.92% to 23.80% ± 1.38% in DU145 cells, and the changes were compensated by AZGP1P2 silencing (Fig. 4F and I). For the in vivo assay, the tumor volumes were drastically diminished by RBM15 silencing in CRPC cells, and the decreases were restored by AZGP1P2 knockdown (Fig. 4J and K).

**Fig. 5.**
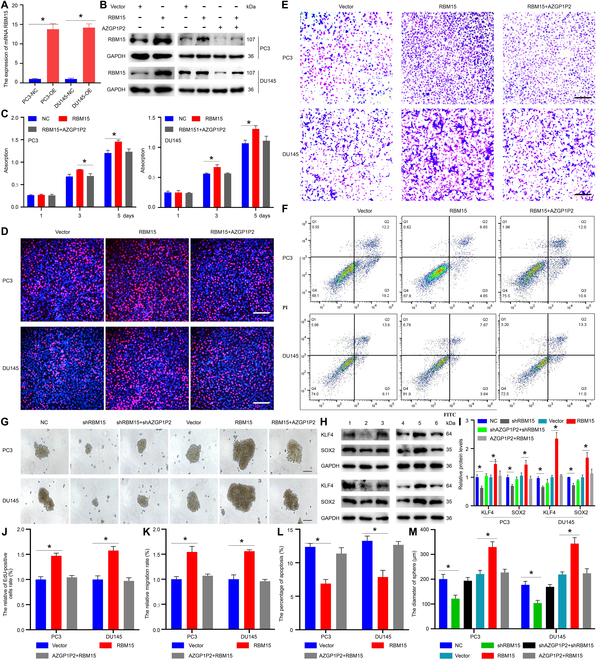
Overexpression of RBM15 promotes the docetaxel chemotherapy sensitivity in CRPC cells. (A and B) Real-time PCR and Western blots were used to reveal the RBM15 overexpression efficiency in PC3 and DU145 cells. (C) CCK-8 assay showed the proliferation of PC3 and DU145 treated with docetaxel after overexpression of RBM15 and AZGP1P2. (D to F) EdU assay and Transwell assay showed the EdU incorporation and migration abilities of PC3 and DU145 cells treated with docetaxel after overexpression of RBM15 and AZGP1P2. Scale bars in D and E =100 μm. (F) Annexin V-FITC/PI assay illustrated the apoptosis of PC3 and DU145 cells treated with docetaxel after RBM15 and AZGP1P2 overexpression. (G) RBM15 knockdown decreased the stemness of PC3 and DU145 cells, whereas RBM15 and AZGP1P2 overexpression remarkably increased the diameters of clone spheres. Scale bars in G =100 μm. (H) Western blots revealed the protein levels of KLF4 and SOX2 in PC3 (top panel) and DU145 (bottom panel) after RBM15 knockdown or overexpression. Lane 1, NC; lane 2, shRBM15; lane 3, shRBM15+shAZGP1P2; lane 4, vector; lane 5, RBM15 overexpression; lane 6, RBM15+AZGP1P2 overexpression. (I) The relative expression of KLF4 and SOX2 in PC3 and DU145 cells by RBM15 knockdown or overexpression after normalization to the signals of their respect loading control. (J to M) The changes of the relative EdU-positive cells, migration rates, apoptotic cells, and the diameters of sphere in CRPC cells by RBM15 or AZGP1P2 knockdown or overexpression. Data were presented from 3 independent experiments. * indicated statistically significant differences (*p* < 0.05).

Meanwhile, recombinant pLenO-GTP-RBM15-overexpression plasmid was utilized to determine the effect of ectogenic RBM15 on docetaxel chemotherapy therapy in CRPC. Real-time PCR and Western blots demonstrated that the mRNA and protein of RBM15 were significantly upregulated by pLenO-GTP-RBM15-overexpression plasmid in CRPC cells (Fig. 5A and B). Moreover, the increase of RBM15 protein could be rescued by overexpression of AZGP1P2 (Fig. 5B). CCK-8, EdU, and Transwell assays revealed that the proliferation and migration potentials were increased by RBM15 overexpression in CRPC cells treated with docetaxel (Fig. 5C to E, J, and K), whereas the percentages of apoptotic cells were decreased by RBM15 overexpression in these cells (12.37% ± 0.51% vs. 6.90% ± 0.60% in PC3 cells; 13.27% ± 0.71% vs. 7.87% ± 1.00% in DU145 cells) (Fig. 5F and L). Additionally, the effect of ectogenic RBM15 rescued the therapeutic effect of docetaxel induced by AZGP1P2 overexpression.

Parallelly, we examined the stemness of CRPC cells after upregulation and downregulation of RBM15. As shown in Fig. 5G and M, the diameter of the sphere was increased (220.67 ± 15.31 μm vs. 329.33 ± 21.55 μm in PC3 cells; 219.00 ± 10.58 vs. 343.33 ± 24.13 μm in DU145 cells) by RBM15 overexpression, while the enhancement was diminished by AZGP1P2 upregulation. On the other hand, the diameter of the sphere was reduced from 200.33 ± 19.01 μm to 121.33 ± 14.50 μm in PC3 cells by RBM15 shRNA, and from 177.33 ± 14.01 μm to 103.67 ± 11.06 μm in DU145 cells, and the reduction was rescued by AZGP1P2 knockdown. Western blots displayed that the levels of KLF4 and SOX2 were remarkably altered by RBM15 shRNA and/or AZGP1P2 shRNA or overexpression (Fig. 5H and I). Taken together, these data indicate that RBM15 decreases the docetaxel chemotherapy sensitivity in CRPC cells.

### Identification of TPM1 as an effector of the AZGP1P2/UBA1/RBM15 cascade in CRPC

**Fig. 6.**
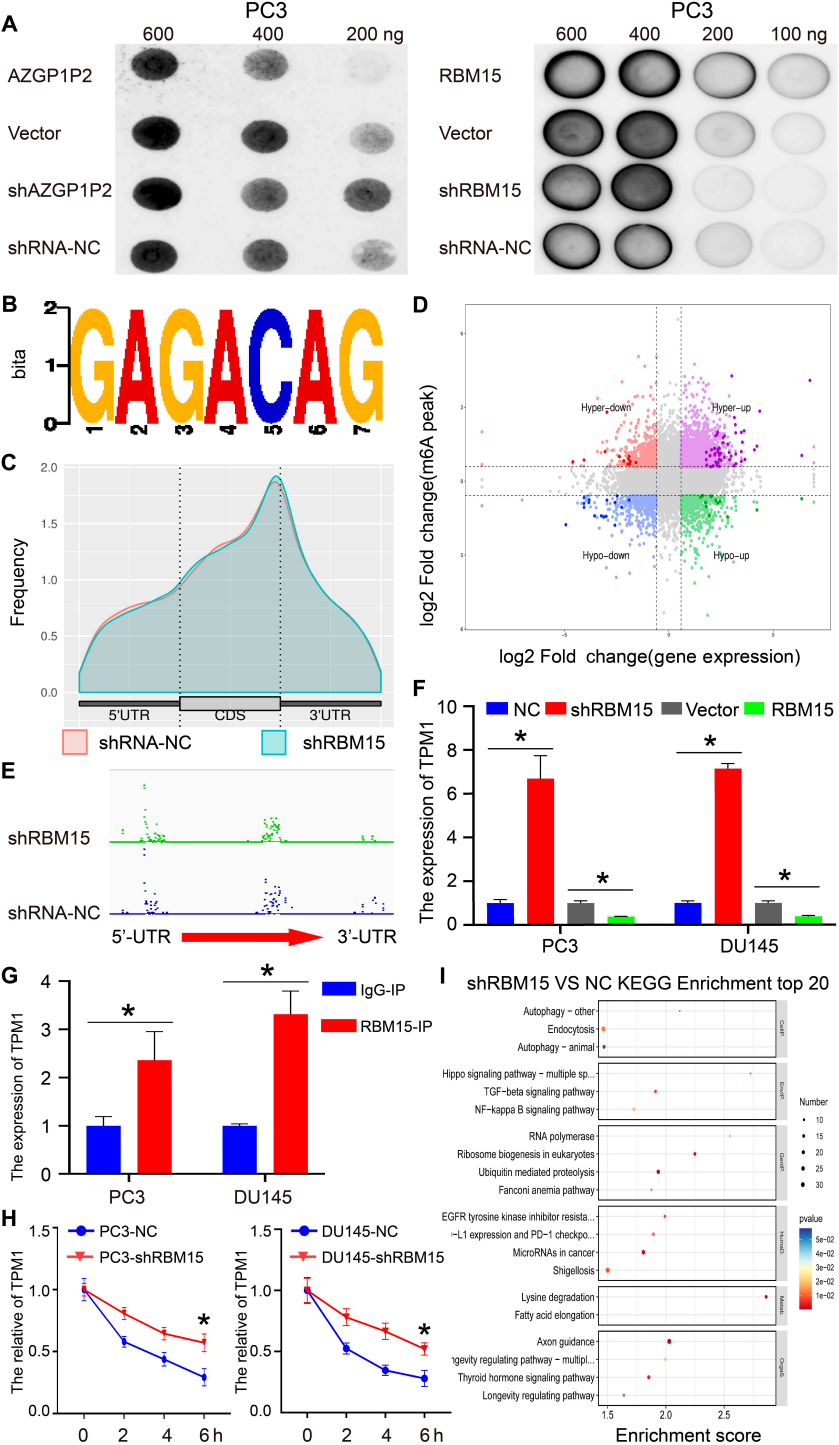
Identification of TPM1 as an effector of the AZGP1P2/UBA1/RBM15 cascade in CRPC cells. (A) Dot blot assay showed that the mRNA m6A levels were positively expressed with RBM15 overexpression and decreased by RBM15 shRNA in PC3 and DU145 cells. (B) The m6A consensus sequence motif was revealed via MeRIP-seq in PC3 cells. (C) MeRIP-Seq analysis showed the m6A peaks distribution at 3′-UTR, 5′-UTR, and coding sequence (CDS). (D) The starplot revealed the genes with differential (hyper or hypo) m6A peaks (*Y* axis; *p* value < 0.05) and differential (up or down) expression (*X* axis; *p* value < 0.05) in RBM15 shRNA compared with the NC group. (E) The m6A modification site of TPM1 mRNA was in the exon region adjacent to 3′-UTR. (F) Real-time PCR showed that the expression of RBM15 was negatively related to TPM1 mRNA. (G) RIP assay further demonstrated the relationship between RBM15 and TPM1 mRNA. (H) Knockdown of RBM15 prolonged the half-life of TPM1 mRNA. PC3 and DU145 cells were treated with actinomycin D (5 μg/ml) for 2, 4, or 6 h before being harvested followed by real-time PCR. (I) Bar plot showed the top 20 enrichment scores of the significant enrichment pathways for the hyper-up, hyper-up, hypo-down, and hypo-down regulated genes. Results were presented from 3 independent experiments. * denoted statistically significant differences (*p* < 0.05).

RBM15 as an m6A writer can recruit the methylation complex of m6A and trigger the methylation of adjacent sites. Dot blot assay showed that, after overexpression of the AZGP1P2, the global level of m6A was substantially decreased (0.80 ± 0.03 at 400 ng of total RNA and 0.61 ± 0.09 at 200 ng of total RNA) in CRPC cells (Fig. 6A and Fig. S4). As expected, the level of m6A was significantly enhanced (1.47 ± 0.05 at 200 ng of total RNA) by shAZGP1P2 in PC3 cells (Fig. 6A and Fig. S4). Besides, the global level of m6A was increased (1.72 ± 0.01 at 200 ng of total RNA, 2.10 ± 0.01 at 100 ng of total RNA) by overexpression of RBM15 in PC3 cells and decreased (0.72 ± 0.06 at 200 ng total of RNA, 0.71 ± 0.02 at 100 ng of total RNA) by RBM15 silencing in PC3 cells (Fig. 6A and Fig. S4). Taken together, our results imply that AZGP1P2 and RBM15 alter the therapeutic effect of docetaxel in PC3 cells in an m6A modification manner. Furthermore, the methylated RNA immunoprecipitation (MeRIP) assay was performed to determine the change of m6A enrichment after downregulation of RBM15. The m6A consensus motif identified in PC3 cells was AGACA (Fig. 6B), which was consistent with a previous finding [22], suggesting that RBM15 can bind to m6A in CRPC cells. As shown in Fig. 6C, m6A peaks were distributed at the area of 5′-UTR, coding sequence (CDS), and 3′-UTR, and mainly surrounding the stop codon area. We conducted a conjoint analysis of MeRIP-seq and mRNA-seq, and a 4-quadrant diagram showed a total of 94 genes (Fig. 6D): There were 53 genes that were increased in MeRIP-seq and mRNA-seq, 16 genes with all decreased levels in MeRIP-seq and mRNA-seq, 13 genes with the decreased levels at m6A and increased levels at mRNA, and 12 genes with a high m6A level and a low mRNA level (Fig. 6D) (Table S5). The m6A-seq analysis demonstrated that RBM15 silencing decreased the m6A accumulation in the exon site of TPM1 (Fig. 6E), which indicates that RBM15 knockdown decreases the level of m6A-modified mRNA. TPM1, a member of the tropomyosin (TPM) family, has been reported as a tumor suppressor gene in numerous kinds of cancers. Furthermore, real-time PCR showed that RBM15 silencing increased the expression of TPM1 mRNA, whereas RBM15 overexpression decreased TPM1 transcript, suggesting that RBM15 promotes the decay of TPM1 in an m6A-dependent manner (Fig. 6F and Fig. S3). To verify the MeRIP-seq results, RIP assay was performed to uncover the target of RBM15. Compared with the immunoglobulin G (IgG) group, anti-RBM15 interacted with TPM1 mRNA at a higher level in PC3 and DU145 cells (Fig. 6G). Indeed, RNA decay rate assay revealed that half-lives of TPM1 mRNA were significantly prolonged by RBM15 knockdown (Fig. 6H). Taken together, these results implicate that the methylation of TPM1 mRNA is recognized by RBM15 and the mRNA stability of TPM1 is increased by RBM15 silencing. In addition, after knockdown of RBM15, KEGG (Kyoto Encyclopedia of Genes and Genomes) analysis of changed m6A peaks showed that NF-κB, TGF-β, and Hippo signaling pathway were significantly changed, and these signaling pathways were associated with CSC destiny (Fig. 6I), which was consistent with previous findings [23–25]. Meanwhile, ubiquitin-mediated proteolysis was activated (Fig. 6I), which further indicates the ubiquitin regulation of RBM15 by UBA1 in CRPC cells.

### AZGP1P2/UBA1/RBM15-TPM1 cascade promotes the chemotherapy sensitivity of docetaxel in CRPC

**Fig. 7.**
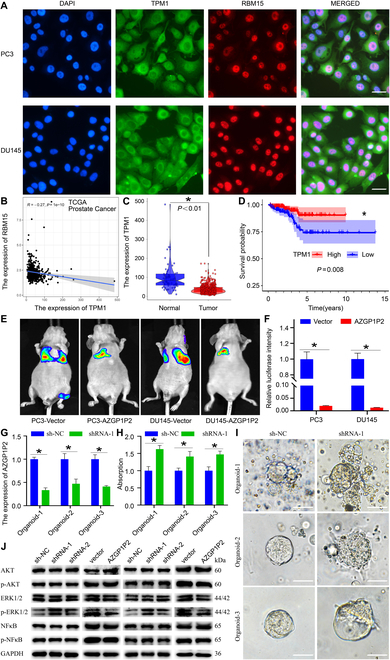
AZGP1P2/UBA1/RBM15-TPM1-ERK1/2 cascade enhances the chemotherapy of docetaxel in CRPC cells. (A) Double immunofluorescence illustrated the subcellular localization and coexpression of TPM1 and RBM15 in PC3 and DU145 cells. Scale bars in A = 10 μm. (B) The correlation analysis revealed the relationship between RBM15 and TPM1 in TCGA database. (C and D) Bioinformatics analysis of the expression and progression-free interval (PFI) of TPM1 in PCa and normal tissues in TCGA database. (E and F) Lung metastases model illustrated that AZGP1P2 enhanced the therapeutic effect of docetaxel in CRPC. (G) Real-time PCR revealed the AZGP1P2 knockdown efficiency in organoids. (H) CCK-8 assay showed the proliferation of organoids treated with docetaxel for 5 days after knockdown of AZGP1P2. (I) Light microscopy showed the volumes of organoid after knockdown of AZGP1P2. Scale bars in I = 100 μm. (J) Western blots showed the changes of phos-AKT, phos-ERK1/2, and phos-NF-κB by AZGP1P2 knockdown. Data were presented from 3 independent experiments. * indicated statistically significant differences (*p* < 0.05).

Double immunofluorescence demonstrated that TPM1 in cytoplasm and nuclei was co-expressed with RBM15 in nuclei of both PC3 and DU145 cells (Fig. 7A). In the TCGA database, Pearson correlation analysis revealed that RBM15 was negatively correlated with TPM1 expression in 499 PCa patients (Fig. 7B), and TPM1 was significantly downregulated in PCa tissues compared with normal tissues (Fig. 7C). The survival curve showed that the PFI rate of PCa patients with a high TPM1 expression was better than that of patients with a low expression (Fig.7D). Furthermore, overexpression of AZGP1P2 decreased the lung metastases of PCa treated with docetaxel as demonstrated by the in vivo tail vein injection mouse model derived from PC3 and DU145 cells (Fig. 7E and F). Furthermore, the proliferation of organoids derived from CRPC with docetaxel resistance patients was enhanced by AZGP1P2 silencing (Fig. 7G to I). Additionally, Western blots demonstrated the changes of phos-ERK1/2 by AZGP1P2 knockdown or overexpression (Fig. 7J). No obvious change of phos-AKT or phos-NFκB was observed AZGP1P2 by knockdown or overexpression (Fig.7J). Collectively, these findings imply that AZGP1P2 promotes the chemotherapy of docetaxel by inhibiting PCa cell growth and migration as well as the metastasis of PCa through the AZGP1P2/UBA1/RBM15-TPM1-ERK1/2 cascade.

## Discussion

**Fig. 8.**
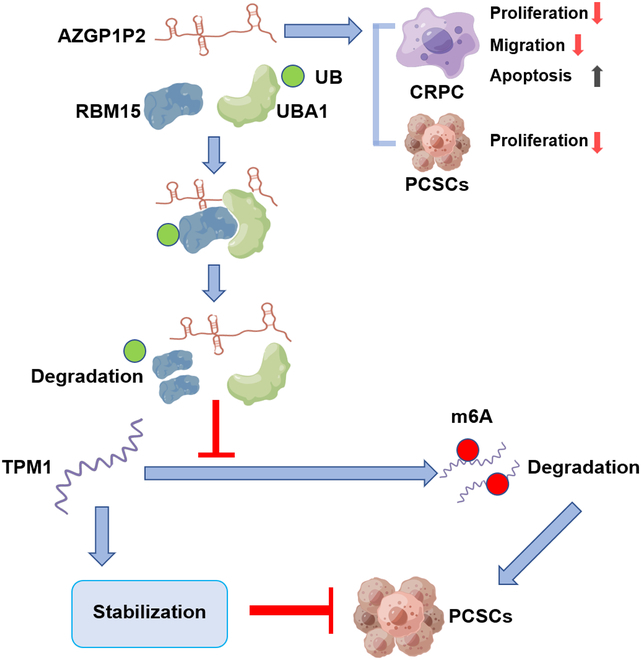
A schematic diagram illustrates the role and molecular mechanism of AZGP1P2/UBA1/RBM15-TPM1-ERK1/2 cascade in the treatment resistance of docetaxel in CRPC. AZGP1P2 can bind to UBA1 and RBM15 in CRPC to form a complex, namely, AZGP1P2/UBA1/RBM15. RBM15 positively mediates m6A and is negatively associated with TPM1. As such, AZGP1P2/UBA1/RBM15-TPM1-ERK1/2 cascade controls the proliferation, stemness, migration rate, and apoptosis of CRPC cells, and significantly, this cascade enhances the chemotherapy of docetaxel in CRPC cells. CRPC, castration-resistant prostate cancer; PCSCs, prostate cancer stem cells.

In this study, we have found that AZGP1P2, acting as a tumor suppressor, was downregulated in CRPC cells, and its low expression level indicated the poor prognosis of PCa. We have demonstrated that AZGP1P2 silencing promoted CRPC cell proliferation and migration and decreased the percentages of apoptotic cells treated with docetaxel by regulating the stemness of CRPC cells. At the same time, we have revealed that AZGP1P2 enhanced the therapeutic effect of docetaxel in lung metastasis via the tail vein injection mouse model and organoid model derived from CRPC with docetaxel-resistant patients. Mechanistically, we have uncovered that AZGP1P2 could bind to UBA1 and RBM15 to form a complex. Notably, UBA1 stimulated the decay of RBM15 in a ubiquitination modification manner, and RBM15 decreased stabilization of the TPM1 mRNA by RBM15-mediated m6A-dependent mRNA decay. In addition, AZGP1P2 increased the chemotherapy sensitivity of docetaxel on CRPC by inactivating the ERK1/2 signal pathway. Collectively, our study unveils a novel function and molecular mechanism by the AZGP1P2/UBA1/RBM15-TPM1-ERK1/2 cascade in the docetaxel treatment resistance of CRPC (Fig. 8).

CSCs, capable of self-renewal and multipotential differentiation abilities, play a critical role in chemotherapy resistance and targeted therapies [26]. PCa displays strong intra-tumor heterogeneity, and there are four CRPC subtypes, including CRPC-AR, CRPC-SCL (stem cell-like), CRPC-WNT, and CRPC-NE [27]. Notably, the CRPC-SCL subtype constitutes the second most prevalent group of CRPC, and it exhibits much shorter time under androgen receptor (AR) signaling inhibitors’ treatment compared to CRPC-AR [27], reflecting that PCSCs contribute to the treatment resistance of the AR signaling inhibitor. We utilized the defined SFM to obtain PCSCs according to the method as previously described [28,29]. Real-time PCR showed that AZGP1P2 was downregulated in PC3 and DU145 stem-like cells compared with parent cell line, suggesting that AZGP1P2 is possibly involved in chemotherapy resistance of docetaxel in CRPC via regulating the stemness of PCSCs. It remains unclear whether AZGP1P2 participates in the docetaxel chemotherapy resistance. In this study, we have demonstrated that AZGP1P2 was expressed at a lower level in PCa cell lines, including PC3, DU145, 22RV1, and Lncap compared to human prostate epithelial cells. Furthermore, the PFI level in high expression of AZGP1P2 patients was better than low-expression-level samples, suggesting that AZGP1P2 participates in the development of PCa as a tumor suppressor gene.

Increasing evidence has shown that pseudogenes are aberrantly expressed in multiple biological processes, and they are involved in multiple biological processes, e.g., signaling pathway regulations, immunological response, and catalytic reactions [30]. We utilized AZGP1P2 silencing and overexpression in PC3 and DU145 cells to explore the function in docetaxel chemotherapy resistance. Interestingly, we demonstrated that AZGP1P2 promoted the chemotherapy sensibility of docetaxel chemotherapy through CCK-8, EdU, Transwell, and Annexin V-FITC/PI assays in vitro. Furthermore, we found that the volumes of subcutaneous tumors derived from AZGP1P2 overexpressing PC3 and DU145 cells were decreased after they were treated with docetaxel in vivo. Taken together, our results imply that AZGP1P2 promotes the therapeutic effect of docetaxel in vitro and in vivo and that AZGP1P2 can be utilized as a promising therapeutic target in human CRPC.

To uncover the underlying mechanism of AZGP1P2 in regulating the sensitivity of docetaxel chemotherapy resistance, Co-IP-MS and Co-IP-Western blots were performed and showed that AZGP1P2 could bind to RBM15 and UBA1 to form a complex to regulate the chemotherapy sensitivity of docetaxel. Furthermore, our RIP assay demonstrated the interaction of RBM15/UBA1 with AZGP1P2. Since UBA1 is a ubiquitin-activating enzyme, we explored the ubiquitin level of RBM15, and our findings suggest that UBA1 increases ubiquitination and proteolytic degradation of RBM15. As such, we have uncovered a novel mechanism by which AZGP1P2 regulates the chemotherapy sensitivity of docetaxel in CRPC.

It has recently been reported that m6A RNA methylation participates in various biological processes and diseases, including cancer progression, cell differentiation, cancer cell stemness, and treatment resistance [31–33]. RBM15, as an m6A reader, promotes m6A modification and determines RNA fate by influencing post-transcriptional regulation, e.g., RNA stability, splicing, and genome stability [34]. Our results showed that RBM15 facilitated the proliferation and migration and decreased the percentages of apoptotic cells treated with docetaxel treatment in PC3 and DU145 cells. Moreover, RBM15 inhibition abolished the increases in the proliferation and migration of PC3 and DU145 treated with docetaxel induced by AZGP1P2 knockdown. It has been shown that RBM15 regulates B cell differentiation and inhibits myeloid and megakaryocytic expansion [35]. On the other hand, RBM15 has been shown to be required for hematopoietic stem cell niche [36]. In the present study, we found that RBM15 promoted the PC3 and DU145 cell stemness that is involved in the treatment resistance of docetaxel.

After silencing or overexpression of AZGP1P2 or RBM15, the globe m6A level was dramatically changed in our dot blot assays, which indicates that AZGP1P2 and RBM15 are involved in the chemotherapy sensitivity of docetaxel in CRPC cells in an N6-methyladenosine-modified dependent manner. MeRIP assay was preformed to further illustrate the role of RBM15 in CRPC. We found that the m6A peak on TPM1 was significantly downregulated by RBM15 silencing in PC3 cells. Furthermore, RIP assay demonstrated that the RBM15 protein could bind to mRNA of TPM1. Taken together, these data suggest that TPM1 is a downstream target of RBM15 in regulating the chemotherapy sensitivity of docetaxel in CRPC, which is consistent with the observation regarding RBM15-dependent m6A post-transcriptional regulation [37]. TPM has been firstly identified as an actin-binding protein with double-chained α-helical coiled coil [38]. Aside from the fact that it stabilizes the cell skeleton, TPM plays an important role in multi-physiologic processes, e.g., cell proliferation, apoptosis, and cell motility [39]. There are 4 subtype genes, namely, TPM1, TPM2, TPM3, and TPM4, in the TPM family in mammals. Previous studies have shown that abnormal expression of TPM induces a variety of muscle diseases and cancer diseases, including tumorigenesis or progression [40,41]. As a member of TPM family, TPM1 has been regarded as a tumor suppressor gene in a series of tumors, e.g., bladder cancer, lung cancer, breast cancer, colorectal cancer, gastric cancer, and hepatocellular carcinoma [42–45]. In PCa, PC3 cell-derived exosomal microRNA-183 has been shown to stimulate their proliferation, migration, and invasion by downregulating the expression of TPM1 [46]. In addition, the high expression level of TPM1 enhances the sensitivity of colorectal cancer cells to oxaliplatin via BCL-2 and BAX [47]. As such, TPM1 may promote the sensitivity of docetaxel via regulating cancer cell stemness in CRPC.

We further utilized the organoids derived from CRPC with docetaxel chemotherapy-resistant patients to determine the effect of AZGP1P2 on docetaxel therapeutic resistance, and we found that the proliferation of organoids treated with docetaxel was obviously increased by AZGP1P2 knockdown. Thus, this study may provide a promising therapeutic target for CRPC patients. The ERK1/2 signal pathway plays a vital role in mediating cancer cell and CSC development [48,49]. Interestingly, we demonstrated that the level of phos-ERK1/2 was significantly changed by downregulation or overexpression of AZGP1P2, reflecting that AZGP1P2 mediates CRPC via the ERK1/2 signaling pathway.

In summary, we have explored, for the first time, the function of AZGP1P2 in mediating the sensitivity of docetaxel in CRPC. We found that AZGP1P2 was downregulated in PCa and PCSCs. Mechanistically, AZGP1P2 combines with RBM15 and UBA1, which promotes the ubiquitination and degradation of RBM15 at the protein level. Subsequently, RBM15 silencing enhances TPM1 mRNA stability in m6A transcriptional level modification (Fig. 8). This study thus provides novel insights into molecular mechanisms underlying the chemotherapy of docetaxel via the AZGP1P2/UBA1/RBM15-TPM1-ERK1/2 cascade, and it offers new targets for gene therapy of CRPC.

## Methods

### Patient tissues and cell culture

The informed consent was obtained from the PCa patients and used for research only. This study was approved by the Ethics Committee of Shanghai Tenth People’s Hospital (No: SHSY-IEC-4.1/20-22/01), and the detailed information of the PCa patients was shown in Table S1.

PC3, DU145, Lncap, 22RV1, and RWPE-1 cell lines were obtained from Shanghai Chinese Academy of Sciences (Shanghai, China). RWPE-1 cells were cultured in Defined Keratinocyte SFM medium (Gibco, USA), while PCa cell lines were cultivated with RPMI-1640 medium (Gibco) supplemented with 10% fetal bovine serum (FBS) (Gibco) and 1% penicillin/streptomycin (HyClone, Logan, UT, USA). PCSCs were grown in serum-free Dulbecco's modified Eagle's medium (DMEM)/F12 medium (Gibco) supplemented with 20 ng/ml epidermal growth factor (Sigma, St. Louis, USA), 20 ng/ml basic fibroblast growth factor (Sigma), 0.4% bovine serum albumin (BSA, Sigma), 5 μg/ml insulin (Sigma), and N2 nutrition (STEMCELL Technologies Inc., Canada) as previously described [28]. The cells were incubated at 37 °C in a humidified atmosphere of 5% CO_2_.

### RNA-FISH

RNA-FISH was conducted using a Ribo FISH kit (Ribobio, Guangzhou, China) according to the manufacturer’s instructions. The probes of AZGP1P2 were designed and synthesized by GenePharma (Table S2).

### Quantitative real-time PCR

Total RNA of PCa cells was isolated using TRIzol in terms of the method as described previously [50]. Total RNA was reverse transcribed (RT) into cDNA using the HiScript III 1st Strand cDNA Synthesis Kit (Vazyme, Nanjing, China), and real-time PCR was executed utilizing the ChamQ SYBR qPCR (quantitative real-time PCR) Master Mix Kit (Vazyme, China) pursuant to the manufacturer’s instructions. The primers of genes (Table S2) were designed and synthesized by Sangon (Shanghai, China).

### Western blots

PCa cells were lysed with RIPA buffer (BiotechWell, Shanghai, China) for 30 min on ice for extracting total protein. Cell debris were cleared by centrifugation at 12,000 *g* for 15 min at 4 °C. The protein concentrations were quantified by the BCA kit (Beyotime Biotechnology, Shanghai, China). A total of 30 μg of proteins per sample was separated in 4% to 12% sodium dodecyl sulfate–polyacrylamide gel (SDS-PAGE) gels (Bio-Rad Laboratories) and transferred onto polyvinylidene difluoride (PVDF) membranes (Sigma-Aldrich). The membranes were blocked with 5% milk for 1 h at room temperature and then immunoblotted with primary antibodies overnight at 4 °C. The detailed information on the primary antibodies was shown in Table S2. After washing with PBS-Tween 20 (PBS-T) 3 times, the membranes were incubated with horseradish peroxidase-conjugated secondary antibodies, including anti-rabbit IgG (Santa Cruz Biotechnology, CA, USA) or anti-goat IgG (Santa Cruz Biotechnology) at a 1:2,000 dilution for 1 h at room temperature. After extensive washes with PBS-T, the blots were detected by chemiluminescence (Cell Signaling Technology) under the Odyssey 2-color infrared laser imaging system (LI-COR Biosciences, Lincoln, NE, USA).

### Transfection of vectors and shRNAs

PCa cell lines were transfected with vectors or shRNAs using Lipofectamine 3000 (Invitrogen, Thermo Fisher Scientific Inc., USA) according to the manufacturer’s instructions. Plasmid and lentivirus expression vector constructs for overexpression of AZGP1P2 and RBM15 were synthesized by genomeditech (Shanghai, China). Short hairpin RNAs (shRNAs) for the indicated gene sequences (Table S2) were designed and synthesized by genomeditech (Shanghai, China). PCa cells were subsequently transfected and selected with puromycin. The transfection efficacy was assessed using qPCR and Western blots. The luciferase was then incorporated into PC3/DU145-AZGP1P2 stable cells.

### Cell proliferation assays

In total, 2,000 cells/well were seeded into 96-well microtiter plates in RPMI 1640 supplemented with 10% FBS overnight, and they were cultured with 20 nM docetaxel (Meilune, Dalian, China). The proliferation potential of PCa cells was detected by CCK-8 assay (Yeasen, Shanghai, China) following the manufacturer’s instruction. Three independent experiments were performed.

### EdU incorporation assay

Cells were seeded into 96-well plates at a density of 1.0 × 10^4^ cells/well in RPMI 1640 supplemented with 10% FBS overnight to allow the cells to attach the plates. The cells were exposed to docetaxel (20 nM) in complete medium for 48 h, and then they were incubated with 20 μΜ EdU (Beyotime biotechnology, China) for 4 h before fixation. EdU assay was conducted according to the manufacturer’s instruction. The percentages of EdU-positive cells were calculated from at least 500 cells, and 3 independent experiments were performed.

### Cell migration assay

Cell migration assay was performed using 8-μm pore size Transwell chambers (Brand, Germany). In brief, 5 × 10^4^ cells with different treatments were harvested and resuspended in 0.2 ml of serum-free medium with 20 nM docetaxel and seeded into upper chambers, while the bottom chamber was filled with 0.6 ml of medium supplemented with 10% FBS and 20 nM docetaxel. After incubation for 48 h, the migrating cells on the surface of the upper chamber were fixed with 70% ethanol for 30 min and then stained with 0.5% crystal violet aqueous for 15 min. Migration abilities were analyzed under a light microscope (Olympus Corporation, Tokyo, Japan).

### Prostasphere formation

In total, 1,000 CRPC cells/well were seeded into 24-well ultra-low-attachment plates in DMEM/F12 medium supplemented with growth factors as mentioned above. After being cultured for 2 weeks, the sphere size and numbers were measured, and spheres were photographed under a light microscopy. The spheres with a diameter of more than 100 μm were considered as stem cell colonies.

### Organoid culture and lentiviral transduction

CRPC with docetaxel chemotherapy-resistant patient-derived organoids was established pursuant to the method as described previously [51,52]. The organoids were cultured with the advanced DMEM/F12 supplemented with 125 ng/ml rhR-spondin-1 (STEMCELL Technologies, 78213.1), 100 ng/ml rhNoggin (R&D Systems, 6057-NG-025), 1 ng/ml rhFGF (R&D Systems, 233-FB-025), rhFGF-10 (R&D Systems, 345-FG-025), 50 ng/ml (Meilunbio, China, MB8218-1), 1×N21 (R&D Systems, AR008), 10 μM Y-27632 (Abcam, Ab120129), 0.5 μM A83-01 (Beyotime Biotechnology, SF7917), 1:100 primocin (Invivogen, ant-pm-1), 10 μM SB202190 (Beyotime Biotechnology, SC0380), 10 mM Nicotinamide (Beyotime Biotechnology, S1761), and 1.25 mM N-acetylcysteine (Selleck, S1623), and the medium was changed every 2 to 3 days. Lentiviral transduction of AZGP1P2 shRNA and shRNA control was conducted according to the method as previously described [53]. The proliferation of organoids treated with 20 nM docetaxel was assessed by CCK-8 assay on the fifth day, and organoids were photographed using light microscopy. Three independent experiments were performed.

### Flow cytometry

Annexin V-FITC/PI KIT (Becton, Dickinson and Company, catalog no. 556547, USA) was utilized to detect the percentage of apoptotic cells. ALDEFLUOR assay (STEMCELL Technologies, Herndon, VA, USA) was performed to examine the stemness of CRPC cells. Treated cells were harvested and washed with PBS twice, and they were incubated with appropriate antibodies on ice following the manufacturer’s instruction. After being washed with PBS, the cells were analyzed on LSRFortessa SORP (BD Biosciences, Franklin Lakes, NJ), and data were analyzed in FlowJo (Ashland, OR).

### Immunocytochemistry and immunohistochemistry

Immunocytochemistry and immunohistochemistry were performed according to the procedure as we previously described [53]. In brief, PCa cells or tissue sections were fixed with 4% paraformaldehyde (PFA) for 30 min and permeabilized with 0.5% Triton X-100 (Sigma) for 5 min followed by washing with PBS adequately. The cells or tissue sections were blocked with 5% BSA (Sigma) for 1 h and then incubated with primary antibodies, including RBM15, UBA1, or TPM1 (Santa Cruz Biotechnology, sc-74480), overnight at 4 °C. After washing 3 times with PBS, the cells or tissue sections were incubated in FITC or rhodamine-conjugated secondary antibodies (Sigma) at a dilution of 1:200 for 1 h at room temperature. DAPI (4,6-diamidino-2-phenylindole) was used to label the nuclei of the cells, and the images were captured with a fluorescence microscope (Nikon, Tokyo, Japan).

### RNA pull-down assay, RIP assay, and Co-IP assay

RNA pull-down kit (Bersinbio, Guangzhou, China) was used to perform AZGP1P2 pull-down assay following the manufacturer’s instructions. In brief, a total of 1×10^7^ PCa cells were lysed and sonicated. The negative control (NC) and AZGP1P2 probes synthesized by GenePharma (Shanghai, China) were employed for incubation with Streptavidin magnetic beads for 30 min at 25 °C to generate probe-coated beads. The detailed information on the sequences was shown in Table S2. After removing nucleic acid, the protein samples and probe-coated beads were incubated for 2 h at 25 °C to pull down RNA binding proteins (RBPs). RBPs were harvested using protein elution buffer followed by eluting the RNAs bound to the beads. MS was performed to identify the differentially expressed proteins. Western blots were performed to identify the AZGP1P2 binding proteins.

For RIP assay, EZ-Magna RIP kit (Millipore, Billerica, MA, USA) was employed to determine the interaction between RBM15/UBA1 protein and AZGP1P2 mRNA, in terms of the instruction of the manufacturer. The qPCR was performed to examine the association of RBM15/UBA1with mRNAs absorbed by the magnetic beads.

For Co-IP assay, cells were harvested in lysis buffer (Epizyme, Shanghai, China) on ice for 30 min. Meanwhile, 50 μl of Dynabeads Protein G (Yeasen, China) was incubated with 5 μg of antibody at room temperature for 1 h. The protein lysate with the beads–antibody complex was incubated at a shaker overnight at 4 °C. After being washed 3 times with lysis buffer, the bound proteins and 10% inputs were detected by Western blots.

### In vivo ubiquitination assay

For ubiquitination assay, 1×10^5^ PC3/DU145 cells were plated into 6-well plates and treated with 10 μM MG-132 (Yeasen, China) to inhibit the protein degradation. Four hours later, the cells were harvested and lysed with RIPA for 30 min on ice. After centrifugation at 12,000 *g* for 15 min at 4 °C, RBM15 antibody at a dilution of 1:100 together with 40 μl of agarose G (Yeasen, China) was added to the samples to be co-incubated overnight at 4 °C on a rotary shaker. After washing 3 times with PBS, the proteins were separated by SDS-PAGE gels and then transferred on a PVDF membrane. The membrane was blocked with 5% milk for 1 h at room temperature and incubated with ubiquitin antibody (Abcam, catalog no. ab140601) overnight at 4 °C. After extensive washes with lysis buffer, immunoblotting was performed to detect the binding proteins and 10% input.

### Protein degradation assay

Cells were incubated with 20 μM cycloheximide (CHX) (Inalco, USA) for the suitable time. The cells were harvested, and Western blots were performed according to the method as described above.

### The m6A dot blot assay

Total RNAs were isolated from cells with various kinds of treatment and denatured 65 °C for 5 min to open the secondary structures. The samples were divided into 600-ng, 400-ng, 200-ng, and 100-ng groups, and they were dissolved in ice-cold saline sodium citrate buffer (Sigma-Aldrich). The RNA samples were loaded on an Amersham Hybond-N^+^ membrane (GE Healthcare, USA) installed in a Bio-Dot Apparatus (Bio-Rad, USA), and then the membranes were ultraviolet-crosslinked for 30 min. After washes with PBST, the membranes were incubated with m6A antibody (1:5,000, abcam, catalog no. ab208577) overnight at 4 °C. Dot blots were analyzed using an imaging system after incubation with horseradish peroxidase (HRP)-conjugated secondary antibody.

### Methylated RNA immunoprecipitation

MeRIP assay was performed using the MeRIP Kit (BersinBio, Guangzhou, China) to explore the enrichment of m6A according to the manufacturer’s instructions. Briefly, 300 μg of total RNA extracted from the shRNA-RBM15 group and the negative control group was broken into about 300-bp fragments. After 10% input was saved, the RNA fragment was immunoprecipitated with the m6A antibody (Abcam, catalog no. ab208577) or IgG incubated with protein A/G magnetic beads in MeRIP reaction buffer for 2 h at 4 °C. The input mRNA and m6A-enriched mRNA were used to establish RNA sequencing libraries and sequenced on a Novaseq sequencer (Illumina, USA) with PE150 strategy. The MeRIP-seq data were analyzed using MeTDiff software [54]. After being purified, the RNA was RT for qPCR.

### The in vivo evaluation of mice with treatment of PC3/DU145 and docetaxel

Animal studies were conducted according to the permission approved by the Animal Care and Use Committee of Shanghai Tenth People’s Hospital of Tongji University (SHDSYY-2014-3028). Four-week-old male nude mice were fed under specific pathogen-free (SPF) conditions, and they were randomly classified into 2 groups, namely, the control group and the PC3/DU145-treated group. In total, 5 × 10^6^/100 μl of PC3/DU145-treated cell suspension transfected with lentivirus was subcutaneously injected into the right inguinal region of mice. After 1 week, mice were treated with docetaxel (10 mg/kg) via intraperitoneal injection twice a week. Tumor sizes were examined using a Vernier caliper every 3 days as follows: volume = 0.5 × length × width. After 1 month, mice were sacrificed and tumors were isolated and weighed. For a pulmonary metastatic model, DU145-vector or DU145-AZGP1P2 cells (1×10^6^) with luciferase were injected into the tail vein of male nude mice at 8 weeks old. After 1 week, mice were treated with docetaxel similar to the subcutaneous xenograft mouse model. The metastatic tumors were observed weekly after 1 month using the in vivo imaging system. The mice were sacrificed when the weight loss was more than 10% compared with the control, and the metastatic foci were detected using the aniView software.

### Statistical analysis

All data were analyzed by Student’s *t*-test or one-way analysis of variance (ANOVA) with GraphPad Prism 7 (GraphPad Prism Software Inc., San Diego, CA) and presented as mean ± SD. Normality and homogeneity of variances were checked prior to conducting Student’s *t*-test or one-way ANOVA, and *P* < 0.05 was regarded as statistically significant. All experiments were performed in triplicate.

## Data Availability

The data present in this article are available from the corresponding authors with the consent.
