# Supplementary material for: AZGP1P2/UBA1/RBM15 Cascade Mediates the Fate Determinations of Prostate Cancer Stem Cells and Promotes Therapeutic Effect of Docetaxel in Castration-Resistant Prostate Cancer via TPM1 m6A Modification
Source: Research (Wash D C). 2023 Oct 17;6:0252. doi: 10.34133/research.0252 (PMC10581371; doi:10.34133/research.0252)
Supplement: Supplementary 1 — Figs. S1 to S4 Tables S1 to S5 [file research.0252.f1.zip › Supplementary Table 2.docx]

**Supplementary Table 2: shRNA sequences, qRT-PCR primer sequences, FISH probes and pull-down probe sequences, and information on primary antibodies**

**shRNA sequences**

| **Genes** | **Sequences** |
| --- | --- |
| shRNA-NC | GATCTGTTCTCCGAACGTGTCACGTTTCAAGAGAACGTGACACGTTCGGAGAATTTTTTC |
| AZGP1P2-1 | GATCCGCCAGGGAGGACATCTTTATCTCGAGATAAAGATGTCCTCCCTGGTTTTTT |
| AZGP1P2-2 | GTGCCTTCTTCCACTACAACTCGAGTTGTAGTGGAAGAAGGCACTTTTTT |
| RBM15-1 | GATCCGCACGAGAATTTGATCGATTTGCTCGAGCAAATCGATCAAATTCTCGTGCTTTTTT |
| RBM15-2 | GATCCAGATTACCTGGTCATGATCATTGTCTCGAGACAATGATCATGACCAGGTAATCTTTTTTT |

**qRT-PCR primer sequences**

| **Genes** | **Sequences (5'-3')** |
| --- | --- |
| AZGP1P2-F | GCCCAAATCCCACACTCAGA |
| AZGP1P2-R | CAGCCTCCTTCCCCTATCCT |
| AZGP1-F | AACCAAGATGGTCGTTACTCTCT |
| AZGP1-R | AACCAAGATGGTCGTTACTCTCT |
| AZGP1P1-F | AGTGAAGACAGGAAGGCTGA |
| AZGP1P1-R | TGGCCTTCTGAACTTGGCTCT |
| RBM15-F | ACGACCCGCAACAATGAAG |
| RBM15-R | ACGACCCGCAACAATGAAG |
| PROM1-F | AGTCGGAAACTGGCAGATAGC |
| PROM1-R | GGTAGTGTTGTACTGGGCCAAT |
| CD44-F | CTGCCGCTTTGCAGGTGTA |
| CD44-R | CATTGTGGGCAAGGTGCTATT |
| KLF4-F | CCCACATGAAGCGACTTCCC |
| KLF4-R | CAGGTCCAGGAGATCGTTGAA |
| TPM1-F | GCCGACGTAGCTTCTCTGAAC |
| TPM1-R | TTTGGGCTCGACTCTCAATGA |
| GAPDH-F | GGAGCGAGATCCCTCCAAAAT |
| GAPDH-R | GGCTGTTGTCATACTTCTCATGG |

**FISH probe sequences**

| **Genes** | **Sequences** |
| --- | --- |
| NC | TCCTTCCAGCCCTTTCTCTCCCTAGC |
| AZGP1P2 | TCACACAAC+TGAGAAGCCAT+TCGCT |

**Pull-down probe sequences**

| **Genes** | **Sequences** |
| --- | --- |
| NC | UUACAGCCCCAGUUACAAGCAACGU |
| AZGP1P2-1 | UCACACAACUGAGAAGCCAUUCGCU |
| AZGP1P2-2 | AGAGAGUGGGACAUUUCCACAAUUA |

**Information on primary antibodies**

| **Antibodies** | **Methods** | **Dilutions** | **Sources** | **Catalog number** | |
| --- | --- | --- | --- | --- | --- |
| CD133 | WB | 1:1000 | CST | | 64326 |
| CD44 | WB | 1:1000 | CST | | 96848 |
| SOX2 | WB | 1:1000 | CST | | 3579 |
| KLF4 | WB | 1:1000 | CST | | 4038 |
| RBM15 | WB | 1:1000 | Abcam | | 244374 |
| RBM15 | ICC | 1:1000 | Abcam | | 244374 |
| RBM15 | IP | 1:100 | Abcam | | 244374 |
| Ubiquitin | WB | 1:2000 | Abcam | | 140601 |
| m6A | Dot blot | 1:5000 | Abcam | | 208577 |
| UBA1 | WB | 1:2000 | Proteintech | | 67198-1-Ig |
| UBA1 | IP | 1:100 | Proteintech | | 67198-1-Ig |
| UBA1 | ICC | 1:200 | Proteintech | | 67198-1-Ig |
| TPM1 | ICC | 1:200 | Santa Cruz | | 74480 |
| AKT | WB | 1:1000 | CST | | 4691 |
| p-AKT | WB | 1:1000 | CST | | 4060 |
| ERK1/2 | WB | 1:1000 | CST | | 4695 |
| p-ERK1/2 | WB | 1:1000 | CST | | 8544 |
| NFκB | WB | 1:1000 | CST | | 6956 |
| p-NFκB | WB | 1:1000 | CST | | 3039 |
| GAPDH | WB | 1:5000 | Abcam | | 8245 |
