# Supplementary material for: AZGP1P2/UBA1/RBM15 Cascade Mediates the Fate Determinations of Prostate Cancer Stem Cells and Promotes Therapeutic Effect of Docetaxel in Castration-Resistant Prostate Cancer via TPM1 m6A Modification
Source: Research (Wash D C). 2023 Oct 17;6:0252. doi: 10.34133/research.0252 (PMC10581371; doi:10.34133/research.0252)
Supplement: Supplementary 1 — Figs. S1 to S4 Tables S1 to S5 [file research.0252.f1.zip › 4. Supplementary Figures and Figure legends.doc]

### AZGP1P2/UBA1/RBM15 cascade mediates the fate determinations of

### prostate cancer stem cells and promotes therapeutic effect of docetaxel in

### castration-resistant prostate cancer via TPM1 m6A modification

## Hong Wang^1,2#^, Ji Liu^1,^^2#^, Xiaojun Zhu^3#^, Bin Yang^1,2*^, Zuping He^4,5*^, Xudong Yao^1,2*^

**Supplementary Figures 1-4 and Figure Legends**

**FIG. S1**

**FIG. S1 The expression of AZGP1 and AZGP1P1 was not significantly changed by AZGP1P2 silencing or overexpression in CRPC cells. A-B.** Real-time PCR showed that the expression of AZGP1 was not changed by knockdown or overexpression of AZGP1P2. **C-D**. AZGP1P1 mRNA level was not significantly changed by knockdown or overexpression of AZGP1P2.

**FIG. S2**

**FIG. S2. The treatment dose of docetaxel was safety *in vivo.*** Hematoxylin-eosin (H&E) staining showed pathological change of the main tissues, including heart, liver, lung, and kidney after being treated with docetaxel *in vivo*. Scale bars = 100 μm.

# FIG. S3

# FIG. S3. RBM15 was negatively expressed with AZGP1P2 and TPM1. A-B. Immunohistochemical staining revealed that the expression of AZGP1P2 was negatively correlated with RBM15, while there was no significant change with UBA1 in xenograft tumor derived from treated PC3 cells. C. Knockdown of RBM15 increased the expression of TPM1 in treated PC3 cells-xenograft tumor, and vice versa. Scale bars = 100 μm.

# FIG. S4

**FIG. S4. The relative expression of m6A level at serial RNA concentration in PC3 cells by AZGP1P2 (left) and RBM15 (right).** * denoted statistically significant differences (*p* < 0.05).
